# A Well Marked Case of Rubber Poisoning Cured by Substituting a Celluloid Plate

**Published:** 1876-01

**Authors:** W. G. Overstreet

**Affiliations:** Greencastle, Ind.


### A WELL MARKED CASE OF RUBBER POISONING CURED BY SUBSTITUTING A CELLULOID PLATE.

BY W. G. OVERSTREET, GREENCASTLE, IND.

This case was that of a young lady who, in 1864, commenced wearing a partial plate of rubber with five teeth, and six years later had a full upper set mounted on rubber, from this time on she was annoyed almost constantly with sore mouth. She decided to have them reset, which was done, making such changes in the plate as would tend to the least irritation of the membrane, and diminishing the size and depth of the air chamber one half, this, with a mouth wash, seemed for a while to have the desired effect, but no permanent cure was established; the inflammation soon returned in a worse form than before, the membrane covering the entire palatine arch being covered with small white pustules. She also has been compelled at times to remove the plate on account of pain. From the time she commenced using the partial piece in 1864, she can't remember a time when her mouth was entirely free from soreness. About four weeks since, I concluded to remount the teeth a fourth time, this time using celluloid for the base; in one week after she commenced using this new plate, I was surprised on examination to find very little if any soreness, and now after wearing the plate one month the inflammation has entirely subsided and her mouth is sound and healthy. Although it has been demonstrated by chemical analysis that rubber is harmless and has no detelerious effect on the mucous membrane in a majority of cases, ten years trial in this case and a complete cure in a few weeks after leaving it off, certainly is convincing proof that *rubber is poisonous* to some mouths at least; and in all such cases I am satisfied a change to celluloid will be beneficial. This is one of many good reasons why celluloid should entirely supersede rubber as a base for artificial teeth.

